# Effectiveness of the BNT162b2 (Pfizer-BioNTech) Vaccine in Children and Adolescents: A Systematic Review and Meta-Analysis

**DOI:** 10.3390/vaccines10111880

**Published:** 2022-11-07

**Authors:** Jewel Maria Sabu, Izza Zahid, Namitha Jacob, Faith O. Alele, Bunmi S. Malau-Aduli

**Affiliations:** 1 College of Medicine and Dentistry, James Cook University, Townsville, QLD 4811, Australia; 2 College of Public Health, Medical & Veterinary Sciences, James Cook University, Townsville, QLD 4811, Australia

**Keywords:** COVID-19, effectiveness, vaccine, children, adolescents

## Abstract

Efforts to control the COVID-19 pandemic have expanded to the vaccination of children and adolescents. This systematic review assesses the utility of the BNT162b2 (Pfizer-BioNTech) vaccine in children and adolescents aged 5–18 years, considering its effectiveness against COVID infection, hospital and intensive care admission and duration of effectiveness after vaccination. Six databases were searched following the PRISMA guidelines. Pooled estimates and 95% confidence intervals (CIs) were calculated using meta-analysis. Fifteen studies were included in the systematic review, while 12 studies were included in the meta-analysis. Evidence suggests that the two-dose vaccination regime provided high effectiveness of 92% (95% CI, 86–96) against COVID infection. Vaccination also conferred high protection against hospitalisation (91%) and intensive care admission (85%). The vaccine was highly protective against the Delta variant of the virus, but showed a lower protection against the Omicron variant. Most adverse effects were transient and mild, commonly including pain at the injection site, fatigue and headache. Current findings are suggestive of waning immunity over time; however, further research is needed to investigate the relevance of booster doses in this age group. In summary, the Pfizer-BioNTech BNT162b2 vaccine demonstrated high levels of protection against COVID-19 infection and its complications while maintaining an adequate safety profile in children and adolescents.

## 1. Introduction

The COVID-19 pandemic caused by the severe acute respiratory syndrome coronavirus 2 (SARS-CoV-2) virus emerged in late 2019, resulting in high morbidity and mortality rates worldwide [1]. According to the World Health Organisation (WHO) 2022 data, there have been 567 million confirmed cases of COVID-19, with mortality rates exceeding 6.3 million deaths [2] Global estimates showed that children and adolescents under 20 years of age accounted for 21% (57.7 million) of the global cases (278 million) and 0.4% (over 15,700) of deaths [3]. Additionally, these estimates for children and adolescents are based on the age- and sex-disaggregated COVID-19 data reported in the COVerAGE database, which accounts for 51% of the global cases and deaths [3]. Furthermore, the global reproduction number (R_0_) of the virus is high (4.08), indicating exponential growth in the number of cases of the disease [4].

While SARS-CoV-2 infects all groups, clinical manifestations of COVID-19 in children and adolescents can be asymptomatic or with milder symptoms than in adults [5]. Nonetheless, in some cases, particularly when there are underlying clinical conditions, the impact on children and adolescents has been significantly severe, with reported cases of severe lung infections, multisystem inflammatory syndrome (MIS-C), intensive care, hospitalisations or death [5,6]. In addition, the pandemic has significantly impacted social interactions among these age groups with school closures, social distancing, isolation and national and local lockdowns [7]. This has many implications for children’s education and social and mental well-being, with children from vulnerable homes being at greater risk [7]. Notably, there has been an increased incidence of children presenting with anxiety, depression and disturbances in sleep and appetite, as well as marked impairment in social interactions [8]. Lockdowns and isolation also come at an economic and mental cost to the children’s caregivers and the family as a whole [9].

Nevertheless, a combination of control measures, including hand hygiene, social distancing and personal protective equipment, are being used to combat this pandemic [10]. A key preventive health measure that has been paramount in reducing infection, morbidity and mortality has been the uptake of SARS-CoV-2 vaccines [11]. According to the WHO, a primary role of vaccination is to reduce transmission [12]. Mathematical modelling of the global impact of vaccination in the first year of vaccination indicated a global reduction of 63% in total deaths [11]. Reduction of virus transmission in the community and curbing of SARS-CoV-2 infections would create fewer disruptions in schooling and the social lives of these age groups. Vaccination is of additional benefit, as it also provides protection against severe disease upon infection and further long-term risk of developing post-COVID complications such as MIS-C [6]. MIS-C is a severe hyperinflammatory condition that has been documented to manifest 2–6 weeks post-symptomatic or asymptomatic infection secondary to an abnormal immune response [13].

While different vaccines have been approved for the control of COVID-19 in the older population, the WHO Strategic Advisory Group of Experts on Immunisation (SAGE) approved BNT162b2 (Pfizer-BioNTech) vaccines for children aged 6 months and older, having met the necessary criteria for safety and efficacy for administration [14,15]. The BNT162b2 vaccines contain the genetic code (mRNA) of the spike protein, found on the surface of the SARS-CoV-2 virus. Once inside the body, the immune system recognises the spike protein and initiates an immune response [16]. The composition of the vaccine for adults and children includes mRNA, lipids, sugar and acid stabilizers [17]. While the composition of the BNT162b2 vaccines is same for adults and children, the recommended dosage is different. According to the latest recommendations published on 18 August 2022, the recommended dose for individuals aged 12 and above is 0.3 mL each (30 µg), while the dose for children between 5 and11 years is 0.2 mL each (10 µg), and for infants and children aged 6 months to 4 years, it is 3 µg, 0.2 mL each [15]. However, prior to these recent recommendations, the Pfizer vaccine was only recommended for children aged 5 years and older, while Moderna mRNA was recommended for individuals 12 years and above [15,18]. 

Due to the urgency required in the production of vaccines to curb the pandemic, the traditional process of vaccine development and approval, which can typically take 10–15 years, has been accelerated with the combination of phases to 12–24 months [19]. While the process appears to be accelerated, the production of the vaccines was partly because these vaccines are mRNA vaccines. The cellular process of creating an immunogenic protein molecule, that triggers the production of specific antibodies takes place inside the human cell in an mRNA based vaccine [20], whereas the traditional protein-based vaccine technology relies on the production of the protein molecule outside of the human cells, resulting in an intrinsically slower process [20]. This difference in vaccine technology has been revolutionary in providing immunity rapidly during the COVID-19 pandemic [20]. Nonetheless, the fast-tracked development of these SARS-CoV-2 vaccines has raised concerns about vaccine safety and efficacy, especially in children [21].

There is a continual need for surveillance and evaluation of vaccine effectiveness and safety in real-world populations, particularly for the protection of younger age groups. Previous systematic reviews of COVID-19 vaccines have focused on adults or the effectiveness of multiple vaccines in children in terms of confirmed and symptomatic infections only [21,22]. To the best of our knowledge, there is no documented systematic investigation of the Pfizer vaccine’s effectiveness against hospitalisation, urgent and emergency care, ICU admissions and death in children and adolescents. We aim to address this gap with a systematic review and meta-analysis of the effectiveness of the Pfizer-BioNTech vaccine in combating the clinical manifestations of COVID-19 in children and adolescents aged 5–19 years. This review focused on children and adolescents aged 5–19 years because the initial approval and recommendation of the use of the Pfizer vaccine was for this age group. Therefore, this review aimed to evaluate the overall effectiveness of the Pfizer-BioNTech vaccine against SARS-CoV-2 (COVID) infection, hospitalisation and intensive care unit admission due to COVID. In addition, duration of effectiveness after vaccination and adverse reactions were evaluated.

## 2. Materials and Methods

### 2.1. Search Strategy

A systematic literature search of six electronic databases (Medline, PubMed, CINAHL, EmCare, Scopus and Cochrane) was conducted from the inception of the databases to June 2022, following the Preferred Reporting Items for Systematic Review and Meta-Analyses (PRISMA) guidelines. MESH terms and keyword combinations were used to identify articles that reported the effectiveness and safety of the Pfizer vaccine in children and adolescents (Table S1).

### 2.2. Eligibility Criteria and Selection Strategy

Studies were included in the review if they were published in English and focused on children and adolescents aged 5–18 years who had received doses of the Pfizer-BioNTech (mRNA BNT162b2) only. Studies were excluded if they involved participants who received other vaccines or presented results of adolescents and children in combination with adults or pregnant adolescents at the time of vaccination. Case series, case reports, letters, commentaries and reviews were also excluded from the review. Three researchers (J.M.S., I.Z. and N.J.) searched and screened the studies under the guidance of F.O.A. and B.S.M.-A. The process was independently repeated by F.O.A. and B.S.M.-A., and any discrepancies in eligibility between the five authors was resolved by consensus. Endnote version 20.3 was used for the screening process.

### 2.3. Data Extraction/Collection

A standardised data extraction form was developed, and data were extracted independently by three researchers (J.M.S., I.Z. and N.J.). The following information was extracted: the basic characteristics of the studies (author, year published, location, study design, vaccination dose status and SARS-CoV-2 variant investigated) and participant details (age, gender and number). Data on systemic and local adverse reactions and vaccine effectiveness were also extracted.

### 2.4. Data Synthesis and Analysis

Extracted data regarding the effectiveness and safety of the Pfizer-BioNTech vaccine against COVID-19 in children and adolescents was systematically synthesised and analysed. Pooled means of vaccine effectiveness (VE) over time and impact on virus variants were calculated. For ease of understanding and analysis, days from vaccination reported in the studies were converted to weeks. Meta-analysis was conducted using R software version 4.2.0 (R Project for Statistical Computing) and forest plots were created. Raw data (number of events such as COVID positive and COVID negative for each vaccination group) from the studies were extracted and used in the meta-analysis. Using a random effects model, the vaccine effectiveness from each study and the pooled estimates (odds ratio) with associated 95% confidence intervals were presented. The pooled estimates for vaccine effectiveness against all COVID infections, symptomatic COVID, hospitalisation and ICU admissions were presented. Odds ratios were converted to vaccine effectiveness, where VE = (1 − OR) × 100%. I^2^ was used to assess heterogeneity between the studies and rated as low (<25%), moderate (50%) or high (≥75%) [23]. Heterogeneity was investigated through subgroup analysis according to the study design. A meta-analysis for the VE from the time of vaccination was not performed due to varying and overlapping reported durations across the studies. Publication bias was assessed for all COVID infections (main outcome) through visual inspection of the funnel plot and Egger’s test.

### 2.5. Quality Appraisal

The Quality Assessment Tool for Studies with Diverse Designs (QATSDD) was used to assess the methodological quality of the reviewed studies. The QATSDD consists of 16 criteria that enable a critical appraisal of the research design of studies [24]. Three criteria items were deemed irrelevant for the evaluation of the included studies and were excluded. Thirteen criteria were each scored on a scale of 0 to 3, with 0 = criterion not mentioned at all, 1 = criterion very slightly mentioned, 2 = criterion moderately mentioned and 3 = criterion fully explained [24]. The total score obtainable for each study was 39 and the scores were subsequently expressed as percentages for easy comparison and grading. Studies were graded as being of low (<50%), good (50–80%) or excellent (>80%) quality. Quality appraisal was conducted independently by three researchers (J.M.S., I.Z. and N.J.) and confirmed by F.O.A. and B.S.M.-A. Any disparities in scoring were resolved by discussion, after which consensus was reached.

## 3. Results

**Figure 1:**
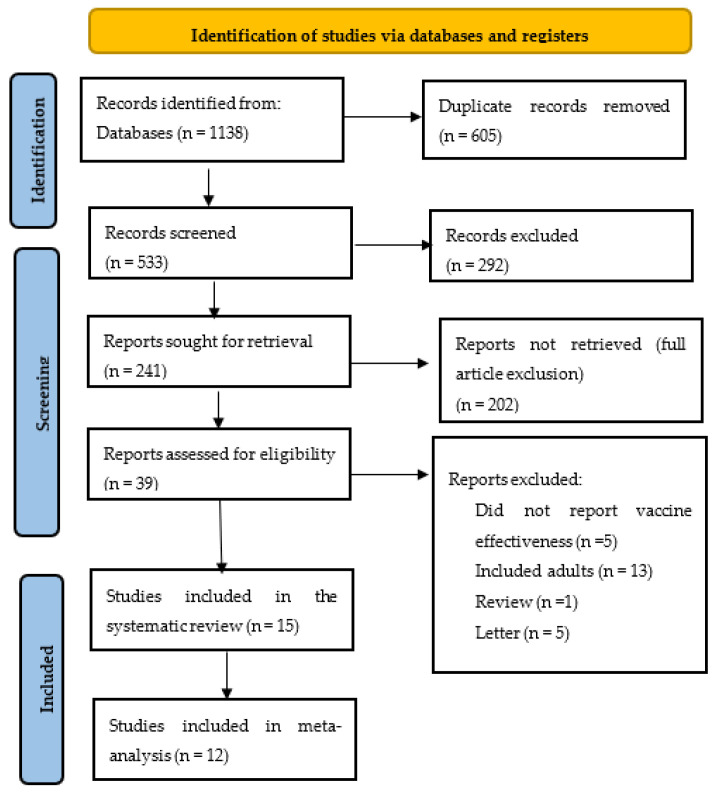
PRISMA flow chart of the systematic review selection process.

As shown in Figure 1, a total of 1138 studies were identified from the databases (Medline, PubMed, CINAHL, EmCare, Scopus and Cochrane), from which 605 duplicates were removed. Upon screening of the titles, abstracts and full texts, 521 articles were excluded. Fifteen (15) studies that met the eligibility criteria were included in the review [25,26,27,28,29,30,31,32,33,34,35,36,37,38,39].

### 3.1. Characteristics of Included Studies

The characteristics of the reviewed studies are presented in Table 1. These comprised six case-control studies [25,31,34,35,36,39], seven cohort studies [26,28,29,30,32,33,37] and two randomised control trials [27,38]. Ten studies originated from the United States of America (USA) [25,26,27,31,32,34,35,36,37,39], one each from Israel [28], South Korea [29] and Denmark [30], and one multinational from the USA, Finland, Poland and Spain [38].

### 3.2. Vaccine Effectiveness against Documented COVID-19 Infection

**Figure 2:**
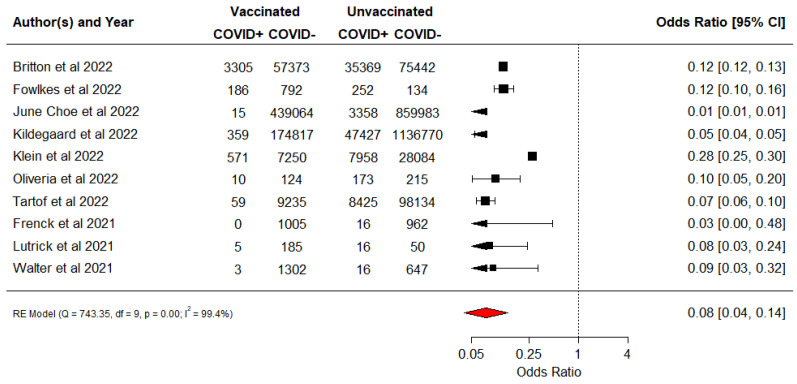
Vaccine effectiveness for all COVID infections [25,26,27,29,30,31,33,34,37,38]. The black square symbol presented for each study represents its point estimate, and the size is proportional to the weight of the study in relation to the pooled estimate. The red diamond symbol represents the overall effect estimate from the meta analysis.

A total of twelve studies assessed VE against COVID infection and reported high VE, with values ranging from 90.7% to 95% [25,26,27,28,29,30,31,33,34,37,38,39]. Two of the studies not included in the meta-analysis reported a VE of 91% and 91.5%, respectively [28,39]. One of the two studies reported VE against multisystem inflammatory syndrome [39]. Meta-analysis (10 studies) showed that vaccination with the BNT123 vaccine was associated with a reduction in COVID infections (Figure 2). The pooled OR was 0.08 (95% CI 0.04–0.14), I^2^ = 99.4%, which corresponds to a pooled VE of 92% (95% CI: 86–96). Sensitivity analysis by study design (Supplementary Figure S1) showed high heterogeneity for the cohort studies (I^2^ = 98.4%) and case-control studies (I^2^ = 99.6%) compared to the randomised control trials (I^2^ = 84.2%).

### 3.3. Vaccine Effectiveness against Documented Hospitalisation

**Figure 3:**
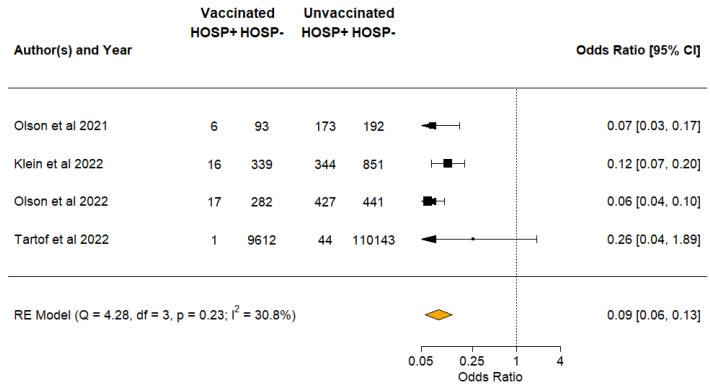
Vaccine effectiveness against hospital admissions [31,35,36,37]. The black square symbol presented for each study represents its point estimate, and the size is proportional to the weight of the study in relation to the pooled estimate. The red diamond symbol represents the overall effect estimate from the meta-analysis.

Four studies evaluated VE against hospital admissions among COVID cases [31,35,36,37]. Meta-analysis of the four studies showed a VE of 91% (pooled OR of 0.09; 95% CI 0.06–0.13) against hospitalisation from COVID infections (Figure 3).

### 3.4. Vaccine Effectiveness against ICU Admissions among COVID Cases

**Figure 4:**
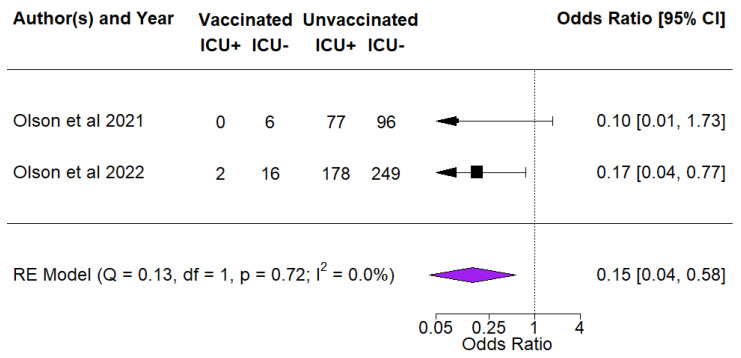
Vaccine effectiveness against admission into ICU [35,36]. The black square symbol presented for each study represents its point estimate, and the size is proportional to the weight of the study in relation to the pooled estimate. The red diamond symbol represents the overall effect estimate from the meta-analysis.

Two studies reported ICU admissions among children and adolescents who had been diagnosed with COVID [35,36]. Findings from the studies showed a VE of 85% (OR = 0.15, 95% CI 0.04–0.5) against ICU admissions among COVID cases (Figure 4).

### 3.5. Vaccine Effectiveness by Time from Immunisation or Vaccination

Seven studies reported the impact of time from immunisation after second dose on the effectiveness of Pfizer-BioNTech [24,25,27,30,31,33,36]. VE was investigated for the Delta strain of the virus in all the studies [24,25,27,30,31,33,36], while only two studies reported VE against the Omicron variant [26,31]. The VE from the studies were pooled and the average VE reported for each week is presented in Table 2. The vaccine was more effective against the Delta variant, with a VE of approximately 91% from 1 week to 12 weeks after vaccination. In contrast, VE against the Omicron variant was 44% within 2 to 20 weeks of vaccination. Overall, VE declined from 13 weeks for the Delta strain and markedly for the Omicron strain from 21 weeks.

### 3.6. Adverse Reactions Associated with Pfizer Vaccine in Children and Adolescents

**Figure 5:**
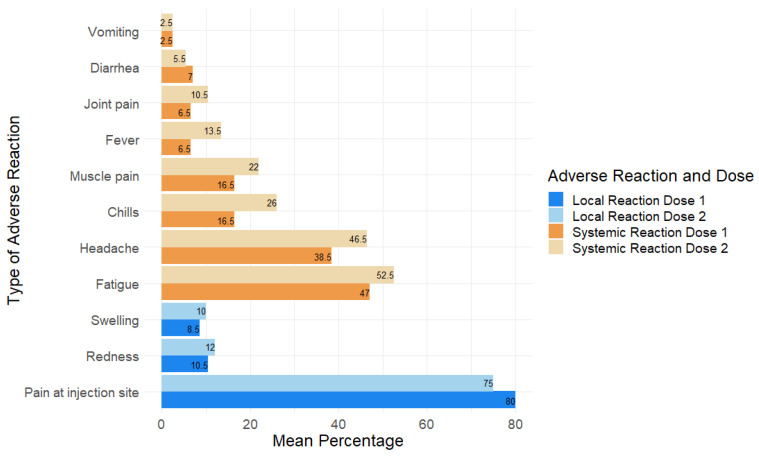
Local and systemic adverse reactions to the vaccine.

Three studies explored the adverse reactions associated with the vaccine [26,28,37]. Given the limited number of studies and heterogeneity in the studies, a meta-analysis was not conducted. Two studies reported local and systemic adverse reactions per dose of the vaccine [27,38], which are presented in Figure 5. Overall, systemic reactions were more common after the second dose of the vaccine compared to local reactions. The most common local reaction was pain at the injection site, which occurred more frequently after the first dose (80% after the first dose and 75% after the second dose). Redness and swelling were the other local reactions reported (Figure 5). The most common systemic reactions were fatigue and headache, occurring at rates of approximately 53% and 47% after the second dose, respectively. Chills, muscle pain, fever, joint pain, diarrhoea and vomiting occurred less frequently. One of the three studies reported rare cases of convulsions, seizures, acute paralysis, anaphylaxis reaction, myocarditis and/or pericarditis [29].

### 3.7. Quality Assessment of the Studies

The studies included in this systematic review were critically assessed for factors that may influence the results’ validity. The aims and/or objectives of each study were clearly stated; although there were variations in the justification of sample size, all the articles defined the study population or people who were recruited into the study. Seven studies in the review controlled for confounders [25,30,32,33,34,36,39]. All the studies except two (which were RCTs) utilised secondary data sources, including registries and hospital records. Therefore, there may be bias associated with the use of these data sources due to inaccuracy of records or other errors. Vaccine efficacy is optimally assessed by means of randomised trials; thus, the inclusion of the two RCTs helps to increase the overall quality of the studies, as a valid comparison will be straightforward. Studies analysing the effects of the vaccine on COVID-19 infection through large-scale cohort studies (seven studies in this review) are also favourable due to obtainable real-world estimates and data. Overall, Table 3 depicts that the quality of the studies ranged from low (33.33%) to good quality (76.92%). Seven studies were rated as low quality [26,27,28,29,31,33,39] and eightstudies as good quality [25,30,32,34,35,36,37,38].

### 3.8. Publication Bias

**Figure 6:**
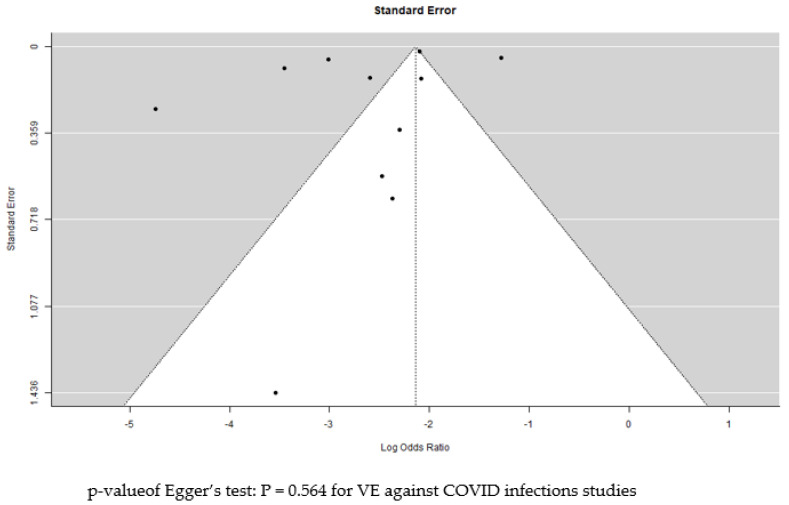
Funnel plot to assess publication bias with Egger’s test—COVID infections. The dots represent individual studies.

Visual inspection of the funnel plot for the VE of the vaccine against COVID infection showed some asymmetry (Figure 6). However, evaluation of publication bias was also assessed using Egger’s regression test, which showed a *p*-value of 0.56, deemed non-significant and indicating no publication bias. The studies included in the other meta-analyses were too few to yield any significant results on publication bias.

## 4. Discussion

This systematic review and meta-analysis investigated the effectiveness of the Pfizer vaccine against COVID infection, hospitalisation and ICU admission. Studies have reported that key influences on parental acceptance of children vaccination included concerns in relation to vaccine adverse effects and beliefs about vaccine effectiveness [40]. In the children and adolescent age group, two key factors that need to be considered are the effectiveness and safety of the vaccine [40].

A key concern about COVID-19 infection in children is that while majority of this population will be asymptomatic or have mild symptoms, there is a subset that may have severe outcomes. Comorbidities in children such as obesity, diabetes, heart disease, lung disease and being immunocompromised have been shown to cause a higher prevalence of severe COVID-19 [41]. Outcomes can include hospitalisation, ICU care, urgent and emergency care or even death [41]. Meta-analysis of VE against hospitalisation and ICU admission yielded high results, thus indicating that vaccination in children and adolescents was protective against severe outcomes of COVID-19. Moreover, there is also the possibility of developing health complications such as MIS-C as a side effect post-COVID-19. The study by Zambrano et al. focusing on MIS-C post-COVID-19 infection found significantly better outcomes for vaccinated persons aged 12–18 than unvaccinated persons, with 91% VE against MIS-C [39]. Ninety-five percent of COVID patients with MIS-C were unvaccinated, with 37% of them requiring life support (respiratory or cardiovascular support) compared to the vaccinated [39].

The reviewed studies that documented VE against COVID infection showed high results after the second dose, and this confirms the value of fully vaccinating this age group (two doses) for optimal protection [25,26,27,29,30,31,33,34,37,38]. As such, a two-dose regime is superior in protecting against confirmed infection (symptomatic and asymptomatic COVID). Previous studies have shown that the mRNA vaccines (Pfizer/BioNTech, NY, USA/Mainz, Germany and Moderna, MA, USA) have satisfactory efficacy ranging from 93% to 100% in preventing COVID infection among children and adolescents, especially the BNT162b2 COVID-19 vaccine [42,43,44]. In contrast, evidence shows that the inactivated vaccine (Sinovac vaccine) has lower efficacy of 66% in children, indicating that the mRNA vaccine is superior in providing protection against COVID infection [43]. While the Pfizer vaccine is highly effective in children, a systematic review and meta-analysis conducted among adults showed that the vaccine effectiveness against COVID infection was 87%, which is slightly lower than the VE reported in our study [45]. The effectiveness against COVID infection is of exceptionally high value in children and adolescents, as infections in this age group are more likely to be asymptomatic than in adults [46]. Some proposed theories for this include age-related endothelial damage, changes in clotting, immunosenescence and a higher prevalence of comorbidities, among others [5]. Therefore, they are more likely to unknowingly spread the infection to others. This makes vaccinating children and adolescents advantageous in reducing the spread of SARS-CoV-2.

While the vaccine was found to be effective overall, its effectiveness waned with time. With the changing pandemic landscape via the emergence of multiple variants of SARS-CoV-2, including the Alpha, Beta, Gamma, Delta, and most recently, Omicron strains, different vaccine effectiveness against different variants have been noted [47]. The evidence from this review showed that the vaccine was highly protective against the Delta variant of the virus, with high VE noted from 1 week to 12 weeks after full vaccination. However, VE wanned subsequently. In contrast, VE against the Omicron variant was weak between 2 and 20 weeks and waned significantly. Studies have reported that VE against symptomatic infection and hospitalisation among children and adolescents for the Omicron variant is low and wanes over time [48,49]. Similarly, existing studies have also reported lower VE for the mRNA vaccines against Omicron in adults in comparison to the other variants [50,51]. The Omicron variant has several sublineages; the main ones are BA.1, BA.2, BA.3, BA.4 and BA.5 [52]. The ability of the sublineages to subvert immunity in fully immunised persons and recovering patients is considered to be a distinctive trait of these sublineages and may explain the lower VE [53]. It is also imperative to note that at the time of this review, there was limited evidence on impact of Omicron among children, with only two studies reporting on VE against this strain. Recent research on Omicron in adults suggests that a booster dose added to the primary vaccination will increase VE against the variant [54]. Vaccine effectiveness over time is important, as this information helps to inform vaccination policies, dose regimes and the need for boosters [55]. A decrease in vaccine effectiveness over time is a typical phenomenon that occurs in most vaccines due to waning immunity [56]. This is consistent with data on decreasing vaccine-derived antibody levels following Pfizer vaccination. This suggests that boosters may be needed in this age group for sustained protection, as evidence shows that booster doses confer additional protection. However, further research on the implications is needed, especially in younger children [48,56].

Additionally, it is important to determine the vaccine’s safety while considering its effectiveness, as acceptance may depend on both factors [40]. In this systematic review, vaccine safety was evaluated by reviewing the adverse events following vaccination. The most commonly reported adverse effects post-vaccination were pain at the injection site, fatigue and headache, all of which were mild to moderate and transient, lasting a few days [27,38]. The more serious adverse reactions were reported only in a very small subset of the population (0.01%) in one study [29]. The incidence of serious adverse effects has also been reported as low in adults, just as indicated for children and adolescents in this study’s findings [57,58].

On an individual patient basis for healthy children and adolescents, in addition to been considered as low risk for severe outcomes, vaccination provides personal protection against post-COVID complications. On a population level, vaccination is more positively associated with reducing the spread to others and improving herd immunity [5,12]. Mathematical modelling of the effect of childhood Pfizer vaccination against SARS-CoV-2 forecasts a high percentage of relative reduction in hospitalisation and deaths, and a small absolute reduction of hospitalisation and deaths, as its incidence is low even in unvaccinated children [59]. Forecasted cases of vaccine-associated myocarditis and anaphylaxis were low [59]. The impact of vaccinating children showed modest herd immunity effects, with a relative reduction in hospitalisations and deaths [59]. The Pfizer-BioNTech vaccine has shown an overall satisfactory safety profile in multiple studies. As such, the benefit-to-risk ratio at both population and individual levels is satisfactory.

A key strength of this review is that it is the first to comprehensively evaluate the Pfizer-BioNTech vaccine’s effectiveness in children and adolescents against the domains of COVID-19 infection, hospitalisation and ICU admission. However, the generalisation of the findings could be limited by the heterogeneity of the reviewed studies, with high I square values in the meta-analyses. This is expected in a systematic review and meta-analysis, which includes studies with different designs and data collection methods. However, when all the studies were pooled and when split by study design, the results from both methods showed high VE, which provides evidence for the validity of the findings. The conducted meta-analysis also increased the quality of evidence and reduced bias. Moreover, the quality appraisal showed that more than half of the reviewed studies were of good quality. Nonetheless, the duration of VE from vaccination varied across studies, indicating that a more specific and defined follow-up period is needed to ensure consistency. Therefore, future studies could investigate the effect of the vaccine on other variants with consistent follow-up times. In addition, 8 of the 12 studies were from the US; while the studies were different, we could not ascertain if subjects have been represented more than once.

## 5. Conclusions

Evaluation of the benefit–risk ratio of the Pfizer-BioNTech vaccine in children and adolescents aged 5–19 years confirms its effectiveness against infection, whether symptomatic or asymptomatic, hospitalisation and ICU, conferring many benefits to vaccine uptake. It also reduces the risk of developing long-term post-COVID complications such as MIS-C. Additionally, vaccination can help reduce the number of cases, ease restrictions and improve the quality of life of people in this age group. The risks of vaccination are relatively small, with non-serious common side effects and a much smaller risk of more serious adverse effects. This confers a satisfactory safety profile to the Pfizer-BioNTech vaccine. Overall, the vaccine is effective against the Delta strain of the virus; however, further research is required to determine its effectiveness against Omicron and other strains.

## Figures and Tables

**Table 1:** Characteristics of the Included Studies.

Author and Year	Country	Study Design	Population Characteristics	SARS-CoV-2 Variant under Investigation
Britton, A. et al., 2022 [25]	United States of America	Test-negative case-control	180,112 adolescents aged 12–19 years (39,422 cases and 140,690 controls). Females accounted for 52.6% of the cases (20,696) and 54.3% (76,108) of the controls, respectively *.	Delta
Fowlkes, A.L. et al., 2022 [26]	United States of America	Prospective cohort study	1364 children and adolescents aged 5–15 years. 1052 were children 5–11 years and 312 were adolescents 12–15 years. Females accounted for 52.3% (713) of the population.	Delta, Omicron
Frenck, R.W., Jr. et al., 2021 [27]	United States of America	Randomised control trial	2260 adolescents aged 12–15 years. Males accounted for 51% of the total population.	Not indicated
Glatman-Freedman, A. et al., 2021 [28]	Israel	Retrospective cohort study	Adolescents aged 12–15 years	Delta
June Choe, Y. et al., 2021 [29]	South Korea	Retrospective cohort study	454,876 12th-grade high-school adolescents	Delta
Kildegaard, H. et al., 2022 [30]	Denmark	Cohort study	991,682 children and adolescents < 18 years of age	Delta
Klein, N. P. et al., 2022 [31]	United States of America	Case-control	39,217 children and adolescents aged 5–17 years. Females accounted for 51.7% (20,310) of the total population	Pre-delta, Delta and Omicron
Lin, D.Y. et al., 2022 [32]	United States of America	Retrospective cohort study ^†^	806,634 adolescents aged 12–17 years *	Delta
Lutrick, K. et al., 2021 [33]	United States of America	Prospective cohort study	243 adolescents aged 12–17 years. Females accounted for 43.6% (106) of the total population.	Delta
Oliveira, C.R. et al., 2022 [34]	United States of America	Case-control	542 adolescents aged 12–18 years. The median age was 14 years (IQR, 13–16 years). Females accounted for 48% (262) of the total population.	Delta
Olson, Samantha M. et al., 2021 [35]	United States of America	Test-negative case-control	464 hospitalised adolescents aged 12–18 years. The median age was 15 years (IQR, 14–17 years). Females accounted for 45.3% (210) of the total population.	Delta
Olson, Samantha M. et al., 2022 [36]	United States of America	Test-negative case-control	1222 adolescents aged 12–18 years. The median age for the cases was 16 years and the median age for the controls was 15 years. Females accounted for 49.5% (605) of the total population.	Delta
Tartof, S.Y. et al., 2021 [37]	United States of America	Retrospective cohort study	201,622 adolescents aged 12–15 years *	Delta, other variants
Walter, E.B. et al., 2022 [38]	United States of America, Spain, Finland and Poland	Randomised control trial	2285 children aged 5–11 years. The mean age was 8.2 years and males accounted for 52% (1182) of the total population.	Not indicated
Zambrano, Laura D. et al., 2022 [39]	United States of America	Test-negative case-control	283 hospitalised adolescents aged 12–18 years. The median age was 14.5 years (IQR 13.4–15.9) and females accounted for 46.6% (132) of the total population.	Delta

* Data related to children and adolescents was extracted from a wider data set that included other age groups. † Study design was determined based on the information provided.

**Table 2:** Vaccine effectiveness from time after vaccination after the second dose.

Duration after Vaccination (Weeks)	Strain	Mean VE
1–4	Delta	91.14
5–8	Delta	91.5
9–12	Delta	91
13–17	Delta	85.3
18–21	Delta	88.4
22–25	Delta	80.2
2–20	Omicron	44
≥21	Omicron	18

**Table 3:** Quality assessment of the studies using the Quality Assessment Tool for Studies with Diverse Designs ( QATSDD).

QATSDD/ Study	1	2	3	4	5	6	7	8	9	10	11	12	13	Total Score	% of Total Score	Grade
Britton et al., 2022 [25]	2	1	2	0	2	3	0	2	0	3	3	1	2	21	53.85%	Good
Fowlkes et al., 2022 [26]	1	0	1	1	1	3	0	0	0	2	1	1	2	13	33.33%	Low
Frenck et al., 2021 [27]	1	2	1	1	1	3	1	0	1	2	2	1	2	18	46.15%	Low
Glatman-Freedman et al., 2021 [28]	0	1	2	0	3	1	0	1	0	3	2	0	1	14	35.90%	Low
June Choe et al., 2021 [29]	2	1	2	0	2	3	1	1	0	2	2	1	1	18	46.15%	Low
Kildegaard et al., 2022 [30]	3	2	3	0	2	2	1	2	1	3	3	2	3	27	69.23%	Good
Klein et al., 2022 [31]	1	1	2	0	2	2	2	2	0	2	2	0	2	18	46.15%	Low
Lin et al., 2022 [32]	2	2	1	0	3	2	1	1	1	3	3	2	2	23	58.97%	Good
Lutrick et al., 2021 [33]	1	1	1	0	1	2	1	1	1	3	2	1	2	17	43.59%	Low
Oliveira et al., 2022 [34]	3	3	3	1	3	3	0	3	0	3	3	3	2	30	76.92%	Good
Olson et al., 2021 [35]	1	2	3	0	2	3	0	2	0	3	3	2	2	23	58.97%	Good
Olson et al., 2022 [36]	2	2	2	1	2	2	0	2	0	3	3	2	2	23	58.97%	Good
Tartof et al., 2021 [37]	3	3	3	1	2	3	1	1	0	3	3	3	2	28	71.79%	Good
Walter et al., 2022 [38]	2	1	0	3	3	3	1	1	0	3	3	3	2	25	64.10%	Good
Zambrano et al., 2022 [39]	1	1	2	0	1	3	0	0	0	2	1	1	2	14	35.90%	Low

1. theoretical framework; 2. aims/objectives; 3. description of research setting; 4. sample size; 5. representative sample of target group; 6. procedure for data collection; 7. rationale for choice of data collection tool(s); 8. detailed recruitment data; 9. statistical assessment of reliability and validity of measurement tool(s); 10. fit between research question and method of data collection (quantitative only); 11. fit between research question and method of analysis (quantitative only); 12. good justification for analytical method selected; 13. strengths and limitations.

## Data Availability

All data generated from the study have been included in the manuscript. Data were obtained from the primary studies included in the review.

## Supplementary Materials

The following supporting information can be downloaded at: https://www.mdpi.com/article/10.3390/vaccines10111880/s1; Figure S1: Sensitivity analysis of VE against COVID infection by study design; Table S1: Medline Search Strategy.
