# Supplementary material for: Effectiveness of the BNT162b2 (Pfizer-BioNTech) Vaccine in Children and Adolescents: A Systematic Review and Meta-Analysis
Source: Vaccines (Basel). 2022 Nov 7;10(11):1880. doi: 10.3390/vaccines10111880 (PMC9698079; doi:10.3390/vaccines10111880)
Supplement: Supplementary file 1 [file vaccines-10-01880-s001.zip › vaccines-1970294-supplementary.pdf]

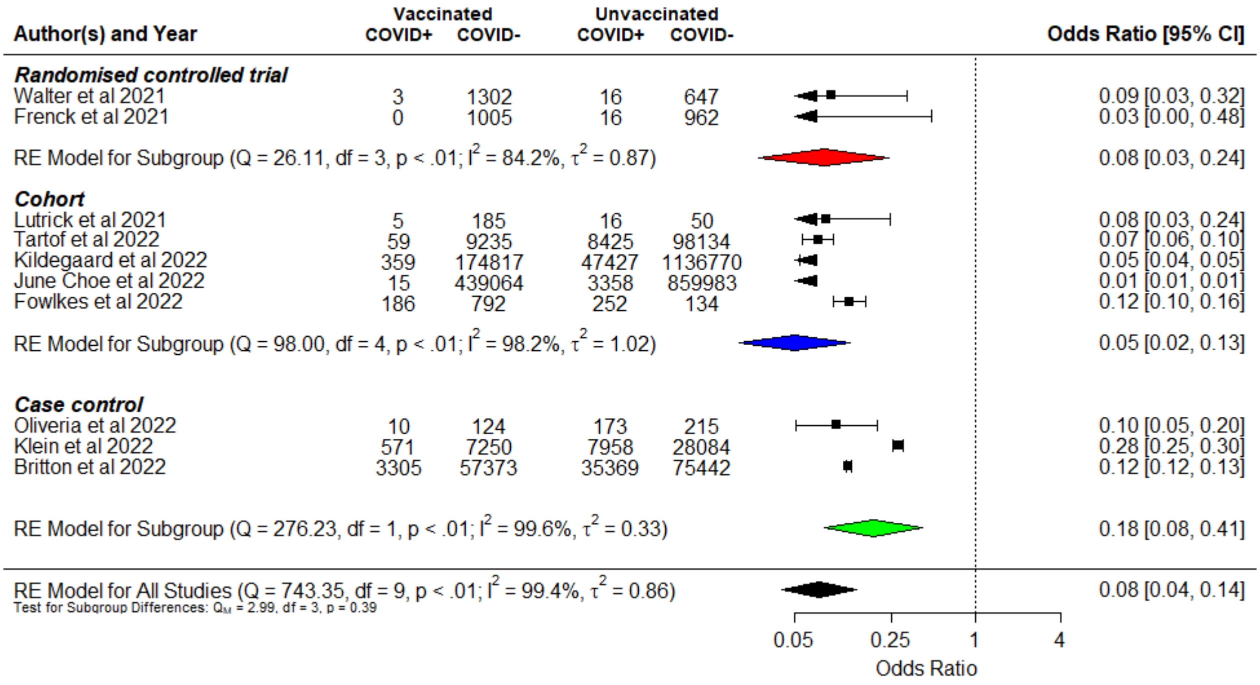

Figure S1. Sensitivity analysis of VE against COVID infection by study design. The black square symbol presented for each study represents its point estimate, and the size is proportional to the weight of the study in relation to the pooled estimate. The red, blue and green diamond symbols represent the overall effect estimate for each study design (randomised control trial, cohort and case-control studies). The black diamond symbol represents the overall effect estimate for all the studies.

Table S1. Medline search strategy.

	Search terms ad combinations
**1**	child OR children OR adolescence OR adolescent* OR "female adolescent*" OR "male adolescent*" OR teen* OR teenager* OR youth*
**2**	bnt162 OR “bnt162 vaccine” OR “covid 19 vaccine pfizer biontech” OR “covid-19 vaccine pfizer-biontech” OR n38tvc63nu OR “pfizer vaccine” OR “Pfizer/BioNTech”
**3**	1 AND 2
